# Sterilising effects of pyriproxyfen on *Anopheles arabiensis* and its potential use in malaria control

**DOI:** 10.1186/1756-3305-6-144

**Published:** 2013-05-17

**Authors:** Caroline Harris, Dickson W Lwetoijera, Stefan Dongus, Nancy S Matowo, Lena M Lorenz, Gregor J Devine, Silas Majambere

**Affiliations:** 1 Vector Group, Liverpool School of Tropical Medicine, Pembroke Place, Liverpool, L3 5QA, UK; 2 Environmental Health and Ecological Sciences Group, Ifakara Health Institute, off Mlabani Passage, P.O. Box 53, Ifakara, Morogoro, Tanzania; 3 Disease Control Department, London School of Hygiene and Tropical Medicine, Keppel Street, London, UK; 4 Current address: Queensland Health, P.O. Box 1103, Cairns, QLD, Australia

**Keywords:** Anopheles arabiensis, Pyriproxyfen, Sterilisation

## Abstract

**Background:**

Insecticide resistance poses a major threat to current vector control campaigns. Insecticides with novel modes of action are therefore in high demand. Pyriproxyfen (PPF), a conventional mosquito pupacide, has a unique mode of action that also sterilises adult mosquitoes (unable to produce viable offspring) upon direct contact. However, the timing of PPF exposure in relation to when mosquitoes take a blood meal has an important impact on that sterilisation. This study investigated the relationship between different blood feeding and PPF exposure timings to determine the potential of PPF sterilisation in controlling *Anopheles arabiensis*.

**Methods:**

Four treatment regimens were investigated: blood fed three days before PPF exposure (A), blood fed one day before PPF exposure (B), blood fed one day after PPF exposure (C) and blood fed three days after PPF exposure (D) for their impact on egg laying (fecundity) and the production of viable offspring (fertility), while the impact of PPF exposure on mosquito survival was investigated in the absence of a blood meal. All regimens and the survival study exposed mosquitoes to PPF via the bottle assay at 3 mg AI/m^2^ for 30 minutes.

**Results:**

Female mosquitoes that blood-fed one day prior to PPF exposure (regimen B), produced no viable offspring during that gonotrophic cycle (100% reduction in fertility). All other treatments had no significant effect. The observed reductions in fecundity and fertility were caused by the retention of eggs (97% of eggs retained, i.e. produced in the ovaries but not laid, in regimen B, p = 0.0004). Some of these retained eggs were deformed in shape. PPF exposure on mosquito survival in the absence of a blood meal was found to have no effect.

**Conclusions:**

The results presented here suggest that sterilising adult malaria vectors using PPF could form part of a malaria control strategy, taking advantage of the lack of reported resistance to PPF in mosquitoes and its unique mode of action. We propose that targeting resting mosquitoes, which are highly susceptible to PPF at low doses, is the optimal direction for developing this control tool.

## Background

Vector control plays a crucial role in combating malaria [1]. The only effective malaria vector control tools widely available are long lasting insecticide treated nets (LLINs) and indoor residual spraying (IRS), both of which use adulticides to reduce vectorial capacity [2,3]. Pyrethroids are the only class of insecticide recommended for treatment of LLINs, while four insecticide classes (organochlorines, organophosphates, carbamates and pyrethroids), which have only two unique target sites, are currently available for IRS [4]. Physiological resistance to all of these classes has already been reported for *Anopheles gambiae* populations with pyrethroid resistance rapidly spreading over recent years [5]. In addition, LLINs and IRS have limitations because they only target indoor biting mosquitoes. As a result of rapidly increasing coverage with LLINs and IRS, *An*. *arabiensis* has become the most abundant malaria vector in many parts of East Africa [6-8]. It blood feeds and rests both indoors and outdoors and current vector control strategies have shown limitations in their ability to control this versatile species [8,9]. In addition, it has also been proposed that the indoor biting and resting mosquitoes; *An*. *gambiae* and *An*. *funestus*, are adjusting their behaviour in response to these interventions by increasing their propensity to feed and rest outdoors and to feed earlier in the evening when people are less likely to be under bed nets [8,10]. These behavioural traits are thought to pose one of the greatest threats to current malaria control programs [11].

One relatively under-exploited class of insecticides are the juvenile hormone analogues (JHAs). Conventionally applied as a pupacide [12-15], their mode of action is different to all the registered adulticides. This class of insecticides interferes with insect morphogenesis, embryogenesis and reproduction [16]. Resistance to PPF has been reported in whiteflies, which are heavily targeted agricultural pests [17,18], but there is no record of resistance in any other insect to date. This and its novel impacts on adult mosquitoes make it a good candidate for combining with other insecticides for vector control [19,20].

In adult mosquitoes, the timing of exposure to PPF in relation to mosquito blood meals is known to affect the mosquitoes ability to produce viable offspring [21] with lifelong sterilisation (unable to produce viable offspring) being the ideal outcome for vector control. A recent study on *An*. *gambiae* concluded that mosquitoes exposed to a 0.1% PPF treated net (35 mg AI/m^2^) for three minutes were rendered sterile for life when mosquitoes blood-fed the night before PPF exposure via a treated bed net [22]. This requires an unlikely scenario in which mosquitoes rest on nets after feeding, therefore more practical methods for exposing mosquitoes to PPF via their host seeking or resting behaviours are required, along with a better understanding of the relationship between the timing of blood feeding and PPF exposure on fertility and fecundity.

This study, therefore, aims to investigate the relationship between time of blood feeding and PPF exposure on *An*. *arabiensis* sterilisation. The effects of PPF on mosquito egg production, egg laying, viable offspring production and survival are explored and discussed in relation to its potential use as a malaria control tool.

## Methods

### Ethical approval

Ethical approval was given by the institutional review board and National Institute of Medical Research (NIMR/HQ/R.8a/Vol. IX/1045).

### Mosquitoes

Mosquitoes from the *An*. *arabiensis* IFAKARA strain colonies were used for all experiments (two independent colonies used, one started in 2007 and one in 2010). The colonies are reared in semi-field systems (SFS) under natural temperature and light:dark cycles for the area. Humidity is artificially increased to around 80% during the dry season, and mosquitoes are fed 10% glucose solution. Routine blood meals for colony maintenance are provided by a human arm daily *ad infinitum*.

### PPF exposure on offspring production

**Figure 1:**
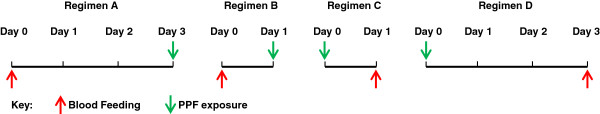
Description of the treatment regimens.

Four different treatment regimens were investigated each with a different length of time between blood feeding and PPF exposure: blood feeding three days prior to PPF exposure (regimen A), blood feeding one day prior to PPF exposure (regimen B), blood feeding one day after PPF exposure (regimen C) and blood feeding three days after PPF exposure (regimen D, Figure 1). Three experimental replicates were conducted for each regimen, each consisted of ten control and ten PPF exposed batches. Each batch consisted of ten unfed female mosquitoes. Each experimental replicate and regimen used mosquitoes from a single cage selected at random. The difference in age of mosquitoes per cage was no more than one day (i.e. pupae emerged in a single night, or two consecutive nights combined). On the day of PPF exposure mosquito age ranged between 5–8 days for regimen A, 5–7 days for regimen B, 5–7 days for regimen C and 5–11 days for regimen D, dependent on experimental replicate. In total 300 PPF exposed and 300 control mosquitoes were used for each regimen.

All mosquitoes were kept under SFS conditions on 10% glucose solution. Any blood feeding event was preceded by a 12 h starvation period. Blood meals were provided by offering a human arm for ten minutes (as per routine colony maintenance). Mosquitoes were exposed to PPF by confining them to treated glass bottles for 30 minutes. Once both blood feeding and exposure were complete, each batch of mosquitoes was maintained in an untreated plastic cup with a net lid and fed *ad libitum* on a 10% glucose solution under SFS conditions. Each day, the numbers of live blood-fed, live unfed, dead blood-fed and dead unfed mosquitoes were recorded. All dead mosquitoes were removed immediately and unfed mosquitoes removed the day after blood feeding.

#### PPF exposure using glass bottles

For all treatment regimens and the survival study, mosquitoes were exposed using glass bottles treated with PPF dissolved in acetone or control bottles treated with acetone alone [23]. One day before exposure, a solution of PPF was prepared by adding 0.25g of pulverised Sumilarv 0.5G (0.5% AI, wt:wt, 5000 ppm, Sumitomo Chemical Co., Osaka, Japan) to 2.5 ml of 100% acetone and agitated overnight. On the day of exposure, 200 μl of that PPF solution (0.1 mg AI) was added to ten 250 ml Schott bottles and an additional 1 ml of acetone was added to aid coverage of the inner surface of the bottle. This resulted in approximately 3 mg AI/m^2^ surface area. In parallel, ten control bottles were prepared using acetone alone. All bottles were capped and rotated in all planes to ensure they were evenly coated. Caps were then removed to allow the acetone to fully evaporate while continuing to rotate the bottles. Each mosquito batch was assigned a corresponding Schott bottle (PPF treated or control) and the mosquitoes were confined to these bottles for 30 minutes. Mosquitoes were then returned to their untreated cups and kept under SFS conditions. This method was adapted from Sihuincha *et al*. [24].

#### Egg production and retention

After the PPF exposure and blood feeding cycle was complete, mosquitoes were held in untreated cups with a damp filter paper as an oviposition substrate. The presence of eggs was recorded daily, and once eggs were present in 50% of the control batches, they were left for a further 24 hours before eggs were counted and transferred to plastic dishes with water covered in netting to allow hatching. Fish food was provided daily and hatched larvae counted and removed from the experiment for four consecutive days. Mosquitoes from one experimental replicate from regimen B were maintained for another four days to determine whether PPF had any delaying effects on the egg laying process.

For one experimental replicate from each regimen, mosquito abdomens were dissected on the day of egg counting to determine the number and characteristics of eggs produced but not laid (retained).

### PPF exposure on mosquito survival

Ten batches of ten, two to three day old, unfed female mosquitoes were exposed to pyriproxyfen by confining them to treated bottles for 30 minutes (as described above). Another ten batches of mosquitoes (control) were confined to untreated bottles. Each of the 20 batches was then transferred to an untreated plastic cup with a net lid and fed *ad libitum* on a 10% glucose solution. All mosquito batches were maintained under SFS conditions and mortality was monitored daily. The experiment was repeated three times.

### Statistical analyses

All statistical analyses were conducted in R v2.12.2 [25] using the lme4 package [26] for linear mixed effects models and coxme package [27] for survival analysis.

#### Reduction in offspring

To measure the effect of PPF on offspring production, the number of larvae per mosquito batch was compared between PPF treated and control batches across all four treatment regimens using a linear mixed effects model with a Poisson distribution and a log link for count data. Initially, the treatment group (control or PPF exposed), regimen and number of mosquitoes per batch at the start of the experiment (which varied around ten), were classified as fixed effects, while mosquito batch and experimental replicate were put in as random effects. As there were the same number of batches as data points, this random variable accounts for the overdispersion observed in the data. Model simplification was conducted by comparing Akaike Information Criterion (AIC) values, which resulted in the minimum adequate model including treatment and regimen with batch as a random effect.

#### Total egg production and retention

For total egg production, the numbers of eggs laid and retained by female mosquitoes were added together to give the total number of eggs produced per batch (laid + retained). A linear mixed effects model was applied for count data as described above. Model simplification was conducted, which resulted in the minimum adequate model including treatment, regimen and the number of mosquitoes per batch as fixed effects and batch as a random effect.

The differences in the proportion of laid and retained eggs between control and PPF exposed mosquitoes were used to determine any significant effects of PPF on egg retention. A linear mixed effects model was fitted with binomial error structure and logit link function for proportion data. The data was fitted to a maximum model including treatment, regimen and the number of mosquitoes per batch as fixed effects and batch as a random effect. Model simplification by comparing AIC values led to the removal of the number of mosquitoes per batch to reach the minimal adequate model.

#### PPF exposure on mosquito survival

A cox mixed effects model (coxme) was fitted to mosquito survival data (number of surviving days) by maximum likelihood. Treatment and experimental replicate were considered fixed effects and batch a random effect.

#### Wing length

To ensure no fitness bias was introduced by non-random assortment of mosquitoes to treatment groups (control or PPF exposed) [28], wing length was compared for a total of 197 PPF treated mosquitoes and 242 control mosquitoes for all live mosquitoes on the day of egg counting across eight experimental replicates and including all four treatment regimens (A, B, C and D) [29]. The Wilcoxon-Mann–Whitney rank sum test was performed on each experimental replicate separately and found no significant differences (all p-values > 0.05), meaning this does not need to be taken into account in all other analyses.

## Results

### Reduction in offspring

**Figure 2:**
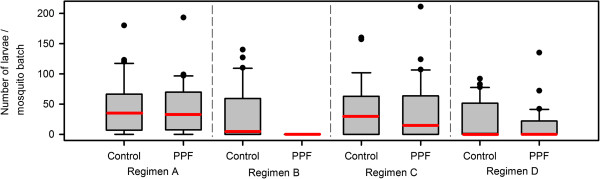
**Effects of PPF exposure on offspring production.** Box and whisker plots (medians: red lines, with inter-quartile ranges) of the number of larvae per batch of mosquitoes exposed to either control or PPF conditions for regimens **A**, **B**, **C** and **D**. Data pooled across three experimental replicates.

Both the number of eggs laid (fecundity) and hatch rates (fertility) were recorded (Table 1). However, it was more meaningful to conduct a single analysis of the total reduction in offspring as the number of eggs laid was close to zero for regimen B (a total of 17 eggs were laid and none hatched from the 300 mosquitoes exposed). Thus, the number of larvae that hatched from each mosquito batch was used as a measure of offspring production to determine any differences after PPF exposure. Regimen B (blood-fed one day prior to exposure, Figure 1) showed complete reduction in offspring, i.e. no viable eggs were produced, whereas no effect was observed for all other treatment regimens (Table 1 and Figure 2).

**Table 1:** **Effects of PPF on*An***. ***arabiensis*egg laying and viable offspring production**

	**Control**			**PPF**			**Comparison offspring production**		
**Regimen**	**Mean eggs laid**	**Mean larvae**	**Mean hatch rate (%)**	**Mean eggs laid**	**Mean larvae**	**Mean hatch rate (%)**	**% reduction in offspring**	**z value**	**p-value**
A	65.8	44.0	66.9	66.1	43.1	65.3	2.0	0.04	0.9691
B	44.4	31.6	71.3	0.6	0.0*	0.0	100.0*	NA	NA
C	50.2	40.6	80.9	40.5	35.4	87.5	12.8	−0.70	0.4868
D	27.9	24.6	88.1	18.6	14.8	79.5	39.9	−0.87	0.3826

Data are from three experimental replicates. The comparison section displays the mixed effects analysis of larval count data, with percent reduction in offspring presented as the result of PPF exposure. * marks where offspring production was 100 percent inhibited.

### Total egg production and retention

**Figure 3:**
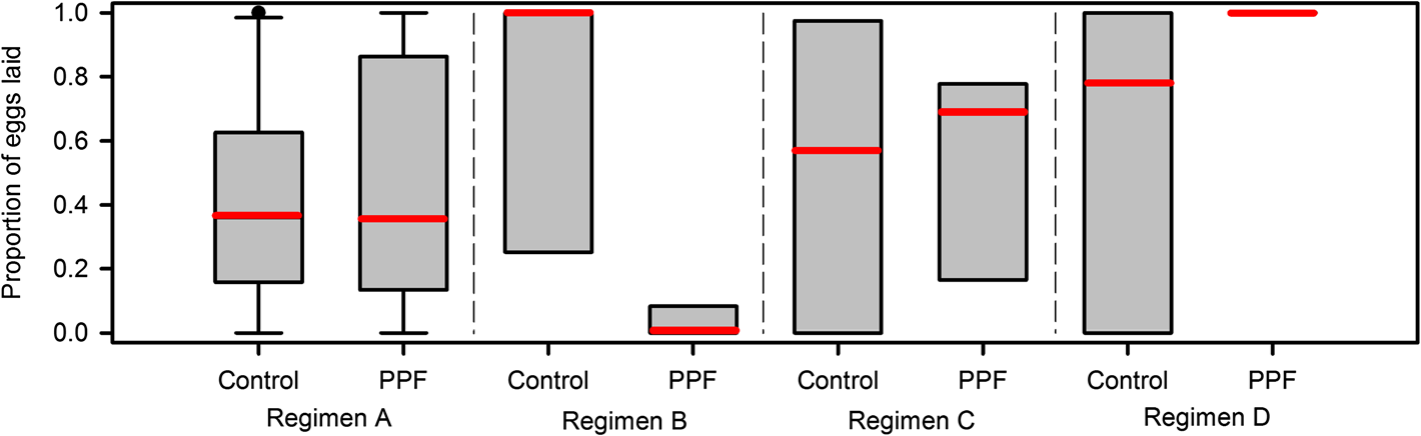
**Effects of PPF exposure on egg laying.** Box and whisker plots (medians: red lines, with inter-quartile ranges) of the proportion of eggs that were laid per batch of mosquitoes exposed to either control conditions or PPF for regimens **A**, **B**, **C** and **D**.

A significant reduction in the proportion of eggs laid was observed for PPF treated mosquitoes in regimen B (p < 0.001) and some of those eggs were deformed. In contrast, regimens A, C and D had no impact on eggs laid (Table 2 and Figure 3). When the number of laid and retained eggs are summed together, all treatment regimens showed no differences in total egg production in comparison to control batches (Table 2). This shows that the reduction in oviposition for regimen B is not due to quantitative differences in oogenesis but simply to egg retention. Total egg production (retained + laid) does appear to decrease from regimen A through to D regardless of whether batches were exposed to control or PPF conditions possibly due to an overall increase in mosquito age used for each subsequent regimen.

**Table 2:** **Effects of PPF on*An***. ***arabiensis*total egg production**, **retention and laying**

	**Control**				**PPF**				**Comparison eggs laid**	
**Regimen**	**Total egg production**	**Mean eggs retained**	**Mean eggs laid**	**Proportion eggs laid**	**Total egg production**	**Mean eggs retained**	**Mean eggs laid**	**Proportion eggs laid**	**z value**	**p-value**
A	2039	118	85.9	0.42	1829	122.7	80.6	0.4	0.6	0.5607
B	793	16.9	82.3	0.83	605	84	2.4	0.03	−3.5	0.0004*
C	513	23.4	79.2	0.77	387	32.8	64	0.66	−0.4	0.6693
D	277	9.7	36.5	0.79	261	0	52.2	1	0	0.983

Means are calculated per mosquito batch. The comparison section displays the mixed effects analysis of the proportion of eggs laid. * marks a significant (p < 0.05) result.

The mosquitoes maintained for another four days after control mosquitoes laid eggs in regimen B showed that no further eggs were laid. Therefore, those mosquitoes that do not lay eggs immediately are completely inhibited from doing so for the whole gonotrophic cycle, rather than the egg-laying process being delayed.

### PPF exposure on mosquito survival

The effects of PPF exposure on mosquito survival while maintained on a sugar diet was tested and found to have no effect (p > 0.05) across all three experimental replicates.

## Discussion

This paper describes the effects of PPF on the sterilisation of *An*. *arabiensis*, one of the most important malaria vectors in many parts of sub-Saharan Africa. The results show that PPF, absorbed via tarsal contact, sterilises mosquitoes for at least one gonotrophic cycle when blood-fed one day prior to PPF exposure, by affecting egg development (in terms of morphology but not numbers) and egg laying. This JHA could be used to sterilise adult malaria vector populations, unaffected by background resistance to other chemicals.

Similar effects on fecundity and fertility have previously been shown in *Anopheles* mosquitoes Miller 1994 unpublished, described in [20,22,30]; however, none have thoroughly investigated the effects of time between exposure and blood feeding. The results presented here clearly show that the time of blood feeding in relation to PPF exposure has significant effects on mosquito offspring production. For regimen A, where eggs were fully developed at the time of PPF exposure, no effect on offspring production (number of hatched larvae per mosquito batch) was observed. In regimen B where mosquitoes were blood-fed one day prior to PPF exposure and thus exposed during the egg development phase, PPF had a profound effect. Most eggs were retained within the mosquito and those which were laid failed to hatch. Neither of the post-feeding regimens had any impact on fecundity or fertility. Studies on *Ae*. *albopictus*, at far higher doses (3 g AI/m^2^) have also shown that exposure after egg development has no effect on eggs laid or larval hatch rate for the immediate gonotrophic cycle [31]. In that species, however, such treatment did reduce the larval hatch rate in the second gonotrophic cycle. This indicates that PPF may remain in the mosquito for some days. A study on *Ae*. *aegypti* mosquitoes, also examining the impact of PPF exposure after egg maturation (equivalent of regimen A), did show a reduction in egg laying [21]. These differences in effect for similar exposure regimens might be due to species-specific differences, mosquito age or the PPF dose used.

In the current study, PPF exposure the day after blood feeding caused complete sterility for that gonotrophic cycle. A previous study on *An*. *stephensi*, using a similar regimen found a significant effect on egg hatching but no effect on the number of eggs laid [30]. This contrasts with the results in the current study where the most striking effects are on egg laying (Table 1). A study on *Ae*. *aegypti* found that the reduction in egg laying was greatest when blood feeding took place after exposure; the opposite of what was found during our study [21]. These differences in the observed responses across species and studies might result from differences in mosquito age or in the exposure method to PPF. It is impossible to compare the potential differential uptake of PPF between the current study method (3 mg AI/m2 on a glass surface) and in the above studies on treated bednets.

The dose-dependent nature of PPF on the nature and duration of observed sterility is confirmed by Ohashi *et al*. [22] who exposed *An*. *gambiae* to 0.1-0.001% PPF treated bed nets (equivalent of 35 mg AI/m^2^ - <10 mg AI/m^2^ of bednet). The highest PPF dose rendered mosquitoes sterile for life when blood-fed the night before exposure (the equivalent to our regimen B). When the dose of PPF was lowered, mosquitoes were sterile for the first gonotrophic cycle but able to lay eggs in subsequent cycles (although still a reduced amount compared to the control). Ohashi’s equivalent of regimen B was highly susceptible to PPF with significant effects on egg laying and hatching even at the lowest doses tested, while their equivalent of regimen C only showed effects on the number of eggs laid [22]. Consistent with the current study, this suggests that mosquitoes are most susceptible to PPF exposure when blood-fed one day prior to PPF exposure and therefore exposed while developing their eggs.

The observed sterilisation was the result of significant egg-retention by affected females. The full impact of retaining eggs, some of which were visibly deformed, requires further exploration. Data from the Ohashi study indicate increased adult mortality after egg retention and this effect is highly dose dependent [22]. The survival analysis conducted in this study shows no effect of PPF exposure in the absence of a blood meal. The effect on survival therefore, seems to only become apparent when the mosquito is exposed to PPF and blood feeds in a manner which affects egg development [22].

The experiments shown here provide new insights into the potential for sterilising mosquitoes via PPF exposure for malaria vector control. Previous studies highlighted its potential use against *An*. *gambiae*[22], while this is the first study to demonstrate similar effects on *An*. *arabiensis*. Although there is a dose dependent effect on the duration over which sterility is observed in *Anopheles*[22], the current study shows that the time of exposure in relation to blood feeding also has a significant impact. Exposing mosquitoes while resting post-blood meal has the greatest effect on fecundity and fertility. This suggests that it will be optimal to target resting mosquitoes. *An*. *arabiensis*, to an extent, evades current control measures due to its limited contact time with insecticide treated surfaces and nets [32]. The potential for mosquito sterilisation with PPF lies in its combination with other vector control strategies that maximise mosquito-PPF contact. The following alternative exposure methods might be used to target *An*. *arabiensis* post blood-meal: treating cow sheds (resting mosquitoes) [33], or treating eave curtains (exiting mosquitoes) [34]. Combinations within conventional interventions such as IRS might still have merit if it could be delivered in a single control measure with dual modes of action, ideally with a non-excito repellent, for insecticide resistance management.

Lifelong sterilization would be required for this to be an effective control measure, either through a single exposure or repeated exposure during each gonotrophic cycle. This will require optimization of the amount of PPF absorbed and therefore the exposure method (determining the contact time) and PPF dose used. As PPF is effective at very low doses there is potential for this to be an effective control method with very short contact times far below the 30 minutes used in the current study. Realistic contact times with treated surfaces inside houses are as low as 2 minutes [35]. Alternatively mosquitoes could physically pick up formulations such as dusts (previously demonstrated for *Aedes aegypti*[36] and *An*. *arabiensis* (Dickson Wilson personal communication) thus extending their exposure times. In doing so, these mosquitoes may also be able to auto-disseminate the PPF to breeding sites during oviposition, a novel pupacidal application method under investigation for malaria vector control. If lifelong adult sterilisation were achieved, these contaminated mosquitoes would ultimately die without reproducing and potentially have reduced survival. It is this effect on survival described in Ohashi *et al*. [22] that will determine whether PPF contaminated mosquitoes are capable of transmitting malaria and mortality prior to potential malaria transmission would offer the greatest outcome. Second to that, if mosquitoes were able to survive long enough to transmit malaria there is still great potential for reductions in vector population densities due to the sterilisation. Mosquito sterilisation with PPF offers a promising new vector control tool to be applied as part of a multifaceted vector control strategy to complement the physical protection and lethal impacts of LLINs and IRS. In doing so, it will help to reduce the spread of resistance, and if applied in a novel manner, target vector populations undeterred by current control measures.

## Conclusion

This study shows that the timing of blood feeding in relation to PPF exposure has important effects on *An*. *arabiensis* sterilisation. Exposure to a dose of 3 mg AI/m^2^ for 30 minutes is sufficient to render mosquitoes infertile for at least one gonotrophic cycle when blood-fed one day prior to PPF exposure, with no effects on all other blood-feeding regimens tested. Resting mosquitoes are thus the most sensitive target for this chemosterilant technique. In order to develop this as a mosquito control tool, the optimal PPF doses and exposure methods must be determined.

## Competing interests

The authors declare that they have no competing interests.

## Authors’ contributions

CH, GJD and SM conceived the study. CH and NSM participated in data collection. CH and LML conducted the statistical analyses. CH, DWL, SD, NSM, LML, GJD and SM participated in its design and coordination and helped to draft the manuscript. All authors read and approved the final manuscript.
